# A Comparative Analysis of Aging Across Causes of Death

**DOI:** 10.1111/eva.70316

**Published:** 2026-09-01

**Authors:** Gabriele Sorci, Bruno Faivre

**Affiliations:** ^1^ Biogéosciences, CNRS UMR 6282 Université Bourgogne Europe Dijon France

**Keywords:** aging, degenerative diseases, infectious diseases, lifespan equality, tissue repair, tolerance

## Abstract

Although there is huge variation in how mortality changes with age across species, in humans, all‐causes mortality increases at a relatively constant rate across populations and time. However, individuals die due to specific causes that include extrinsic and intrinsic sources. Here, we compared the rate at which mortality increases with age (i.e., the rate of aging) for the causes of death supposedly referring to extrinsic (infectious and parasitic diseases) and intrinsic sources (cardiovascular diseases and malignant neoplasm) in 12 human populations. Given that individuals are exposed to infectious agents across their entire lifespan, it is straightforward to expect a shallower rate of aging for infectious compared to cardiovascular diseases and malignant neoplasm. However, infectious and noninfectious diseases might target the same tissues/organs and tissue‐specific repair mechanisms may strongly affect the disease severity. We, therefore, also compared the rate of aging for transmissible and nontransmissible diseases targeting the same tissues/organs. Contrary to the pattern observed when comparing infectious and noninfectious diseases overall, we found that diseases with shared target organs had similar rate of aging, whatever their etiology. We went a step further by assessing the changes in the distribution of cause‐specific mortality across ages (i.e., lifespan equality), between 1955 and 2020, and by investigating whether they were accounted for by changes in early‐life mortality, adult mortality or the rate of aging. This analysis showed that temporal changes in lifespan equality were essentially driven by changes in early‐life mortality, with modest or almost no contribution from changes in adult mortality or the rate of aging. Overall, these results show that the rate at which mortality increases with age is relatively invariant for diseases targeting the same tissues/organs, suggesting that shared tolerance mechanisms, independently from the etiology of the disease, might be important drivers of age‐dependent mortality schedules.

## 1 Introduction

Aging is associated with a series of systemic changes in major physiological functions (López‐Otín et al. 2023), resulting from departures from homeostatic values in molecular and cellular mechanisms (Gladyshev et al. 2021; Kroemer et al. 2025). In this framework, aging can be viewed as a network of cascading effects: age‐dependent mutations and epigenetic changes alter molecular and cellular pathways, leading to impaired physiological functions that ultimately affect the two components of Darwinian fitness: age‐dependent reproduction and age‐dependent mortality (Zhang et al. 2017, 2026; Levine et al. 2018; Li et al. 2023; Tyshkovskiy et al. 2023; Koch et al. 2025). Although not all physiological functions age at the same rate or begin to decline at the same age (Belsky et al. 2015; Nie et al. 2022; Shen et al. 2024), the rate at which mortality increases with age is fairly constant across populations of humans and nonhuman primates (Vaupel 2010; Colchero et al. 2021). This suggests that the decline of different physiological functions does not equally contribute to the age‐dependent increase in mortality. However, mortality arises from specific causes that can be decomposed into intrinsic and extrinsic factors (Carnes and Olshansky 1997; Carnes et al. 2006; Koopman et al. 2015). Intrinsic causes of death are usually defined as mortality arising from an internal failure of bodily functions, whereas extrinsic causes of death require an external trigger. In humans, classical examples of intrinsic and extrinsic causes of death include inherited genetic diseases such as spinal muscular atrophy and Tay—Sachs disease (intrinsic) (Maegawa et al. 2006; Mercuri et al. 2018), and traffic accidents (extrinsic). This distinction is not merely semantic, as the extrinsic mortality regime organisms are exposed to is thought to play a central role in the age‐dependent distribution of intrinsic mortality, that is, how and when organisms age. Indeed, all evolutionary theories of aging share the assumption that the strength of natural selection declines with age (Hamilton 1966). Even in a hypothetical population where organismal performance does not decrease with age, the strength of natural selection declines because environmental hazards limit the number of individuals that ever reach old age. Consequently, late‐age deleterious mutations that have not been eliminated by natural selection can manifest [mutation accumulation theory of aging (Medawar 1952)]. The pattern of extrinsic mortality is also key to the disposable soma theory of aging (Kirkwood 1977). When the risk of mortality due to environmental hazards (e.g., predation) is high, it is not worthwhile to invest resources in anti‐aging mechanisms because the likelihood of dying from causes other than age‐dependent organismal failure is greater. Therefore, when extrinsic mortality risk is high, natural selection is supposed to favor high investment in early reproduction at the expense of soma maintenance (Kirkwood and Rose 1991).

It is important to note that intrinsic and extrinsic mortality are not synonymous with age‐dependent and age‐independent mortality. On the one hand, intrinsic mortality refers to organismal failure and may occur at an early age, as in the case of inherited genetic diseases. Conversely, extrinsic mortality, despite requiring an external trigger, may cluster in specific age groups due to age‐dependent behaviors that amplify the risk. Furthermore, classifying causes of death as extrinsic or intrinsic can be more difficult than anticipated, as in many cases mortality results from the combined effect of external triggers and the organism's inability to repair the resulting damage. In this respect, infectious diseases are a very interesting and paradigmatic example. Infection and predation are often cited as the clearest examples of extrinsic causes of death. Indeed, both require the encounter with an external trigger (a pathogen or a predator). However, unlike predation, where the outcome of the encounter is often instantaneous (the predator either fails to catch the prey or kills it), the outcome of infectious diseases also depends on intrinsic host factors, such as the capacity to resist the infection (i.e., limit the proliferation of the pathogen and clear it) or limit the damage caused by the infection (i.e., repair damaged tissues and restore functions) (Read et al. 2008). Therefore, unlike predation, the outcome of an infection does not depend solely on “bad luck” in encountering a pathogen, but also on the host's intrinsic capacity to deal with the infection, which can be age‐dependent (Simon et al. 2015; Nikolich‐Zugich 2018; Sorci et al. 2021).

In human populations living in high‐income countries, the mortality burden due to infectious diseases has progressively decreased over the last century (Barrett et al. 2024). This epidemiological transition (Omran 2005; Gulis et al. 2025) has occurred because of the reduction of the probability of encounter with pathogens and parasites through improved sanitation/hygiene and vector control, and the enhancement of defenses through antimicrobial drugs and vaccines that act as surrogates of the immune system by limiting pathogen proliferation. The reduction in the mortality burden due to infectious diseases has been accompanied by a concomitant increase in the mortality share due supposedly to intrinsic causes of death, such as cardiovascular diseases and malignant neoplasms (Corbett et al. 2018). However, growing evidence indicates that these pathologies also have environmental triggers, as exposure to soil, water, and air pollution or lifestyle habits (smoking, diet) contribute to the increasing burden of cardiovascular diseases and cancer, adding a strong environmental (extrinsic) determinant to chronic nontransmissible diseases (Ding et al. 2022; Münzel et al. 2023). Therefore, both infectious and noninfectious causes of death have extrinsic (environmental) and intrinsic (e.g., age) triggers.

Causes of death can also share another characteristic: they may affect the same tissues or organs. Interestingly, diseases with specific tissue or organ tropism can exhibit similar pathophysiologies, even when their etiologies differ. For example, both infectious and noninfectious insults to the respiratory system can lead to a common pathological phenotype known as acute respiratory distress syndrome (ARDS) (Ware and Matthay 2000; Matthay et al. 2019), which is characterized by an inflammatory cascade that damages the lung epithelium, disrupts the alveolar‐capillary barrier (causing edema), and results in hypoxemia. Although the incidence of this syndrome is not strongly age‐dependent, its outcome is: old age is strongly associated with increased ARDS mortality. The poor prognosis in the elderly is linked to a reduced capacity to repair damaged epithelium and restore function (Brown et al. 2020). Thus, an important determinant of disease severity is the balance between damage (intensity, rate of accumulation) and repair (resolution of inflammation, restoration of the lung epithelium) (Lucas et al. 2020). Failure of tissue repair mechanisms may therefore account for age‐dependent mortality risk for diseases targeting specific organs, regardless of disease etiology. In other words, tolerance mechanisms shared by the same tissues or organs (e.g., restoration of lung epithelial integrity) may help determine the schedule of age‐dependent mortality for diseases of the respiratory system, independent of their etiology.

In a recent article (Sorci and Faivre 2022), it was shown that mortality (case fatality rate, CFR) from major human infectious diseases generally follows a similar trend as overall mortality, with high CFRs at young and old ages and the lowest CFR at intermediate ages (although this pattern was not consistent across all infectious diseases). Since CFR was analyzed, the sample was restricted to the population of infected individuals. In the present study, we aimed to extend this analysis by examining the age‐dependent mortality schedule in the general population. Specifically, we compared how fast mortality increases with age (i.e., the rate of aging) for major causes of death using mortality data from 12 high‐income countries. While many studies have examined the differences in mortality rates across countries or over time, few have focused on specific causes of death or used a demographic approach to compare rates of increase (see for instance Juckett and Rosenberg 1993; Goldstein and Lee 2020; Sasson 2021). We investigated whether the rate of aging differs between infectious diseases (considered as a broad category) and the two other major causes of death (cardiovascular diseases and malignant neoplasm), while controlling for baseline adult mortality rate. As individuals are exposed to infectious agents throughout their lives, we expected to find that the rate of aging for infectious diseases is shallower than for cardiovascular diseases and malignant neoplasms. Then, we examined whether the rate of aging varies between infectious and noninfectious diseases that target the same tissues or organs. Here, we predicted that if tissue‐specific repair mechanisms are important in shaping the age‐dependent mortality schedule, the rate of aging should be similar for causes of death involving the same tissues or organs, regardless of the disease's cause. Finally, we assessed the temporal changes in the distribution of cause‐specific mortality across ages (i.e., lifespan equality) and identified the underlying causes as changes in early‐life mortality, adult mortality, or the rate of aging. Here, we predicted that lifespan equality would increase over time due to a more homogeneous mortality schedule in old age, particularly with regard to infectious diseases. We also predicted that the main driver of this change would be the sharp reduction in early‐life mortality.

## 2 Methods

### 2.1 Data

We used cause‐specific mortality rates reported in the World Health Organization (WHO) mortality database (https://www.who.int/data/data‐collection‐tools/who‐mortality‐database). Only a few countries contributed data starting in 1950, with the number of entries increasing in subsequent years; therefore, we used data from 1955, when the epidemiological transition was likely not yet completed. Although the database only includes mortality data with at least 65% completeness, we focused on a short‐list of high‐income countries, which likely provided higher quality mortality records from the outset. In the database, age‐dependent mortality is reported as both the number of deaths and as mortality rate per 100,000 for age classes from 0 to 85+ years (the last being an open class). Although the database provides mortality data separately for females and males, we analyzed both sexes combined to ensure sufficiently large numbers of deaths per age class for reliable mortality rate estimates. Therefore, we preferred to have better mortality estimates for both sexes combined, even though this ignores potential differences in mortality schedules between women and men.

Causes of death are attributed as “the disease or injury which initiated the train of morbid events leading directly to death according to the International Classification of Diseases (ICD)”, with only medically‐certified deaths being reported (WHO Mortality Database 2025). The ICD includes very detailed causes of death; however, we preferred using broad categories as to avoid small numbers of deaths per age class and to have consistent disease reports across different versions of the ICD (ICD‐7 to ICD‐10). We therefore focused on three main causes of death: infectious and parasitic diseases, cardiovascular diseases, and malignant neoplasm.

We also wished to compare causes of death that have different etiologies but similar disease tropism (i.e., targeting the same tissues/organs). To this purpose, we focused on diseases that target the respiratory system having either an infectious etiology (e.g., influenza and related viral infections) or a noninfectious etiology (e.g., chronic obstructive pulmonary disease), and on diseases that target the digestive system having either an infectious etiology (e.g., bacterial and protozoal intestinal infections) or a noninfectious etiology (e.g., noninfectious enteritis and colitis).

Given the uncertainty on the completeness and the accuracy of the attribution of the cause of death of the 1955 data, we repeated the analysis (using the same countries) with the 2020 mortality data (with 100% completeness and precise cause of death attribution, https://platform.who.int/mortality/about/data‐quality). However, for the 2020 data, we could not run the comparison between infectious and noninfectious digestive diseases since the mortality due to diarrhoal diseases dropped to very low numbers (including zeros).

Table S1 in the Supporting Information reports the list of countries and the categories of diseases included in the analyses. All data are publicly available on the WHO website (https://www.who.int/data/data‐collection‐tools/who‐mortality‐database).

### 2.2 Estimating the Rate of Aging for Specific Causes of Death

The rate of aging was assessed by computing the parameter *b* of the Gompertz mortality model for each country and cause of death. From adult age onwards, mortality rates can be fitted using the two‐parameter Gompertz model:

(1) $$\mu_{x}=ae^{\mathrm{bx}}$$

where *μ*_*x*_ is the mortality rate at age *x*, *a* is the initial mortality and *b* is the exponential increase of mortality with age (i.e., the rate of aging). The model can be linearized by using log mortality rate:

(2) $$\ln\left(\mu_{x}\right)=\ln\left(a\right)+\mathrm{bx}.$$

This model was run over the 30–85 (5‐year) age classes, using mid values per age class (i.e., 32.5, 37.5, etc.). We did not use the data for the 85+ age class, to avoid problems associated with mortality compression in this open‐ended interval (Kannisto 2000; Ediev 2018). However, excluding the 85+ age class should only marginally affect parameter estimates and, more importantly, should not bias comparisons between causes of death because the same truncation was applied consistently across all analyses.

The *b* parameter of the Gompertz model is commonly used to compare the rate of aging between different populations or species (Reznick et al. 2004; Hawkes et al. 2009). However, a possible drawback in using *b* for comparative purposes is its strong covariance with *a*, known as the Strehler–Mildvan correlation (Burger and Missov 2016), which can make it difficult to distinguish genuine differences in the rate of aging from differences in adult baseline mortality. To address this issue, we centered the age values, which reduces the within‐model covariance between *a* and *b* (Table S2). The fits of the different Gompertz models are shown in Figures S1 and S2 of the Supporting Information.

To compare the rate of aging between causes of death, we ran linear mixed models (LMM) with the following structure:

(3) $$b_{\mathrm{ij}}=\alpha +\beta_{1}c_{\mathrm{ij}}+\beta_{2}a_{\mathrm{ij}}+u_{j}+\epsilon_{\mathrm{ij}}$$

where *b*_*ij*_ is the rate of aging for country *i* and cause of death *j*, *c*_*ij*_ is the cause of death, *a*_*ij*_ is the log adult mortality rate at centered age, $u_{j}\sim N\left(0,s_{u}^{2}\right)$ is the random intercept for country, and $\epsilon_{\mathrm{ij}}\sim N\left(0,s_{e}^{2}\right)$ is the distribution of errors. Although centering age in the Gompertz models disrupts the within‐model covariance between *a* and *b*, this does not guarantee the lack of covariation between the two parameters across countries. Therefore, *a* was included in the LMM as a fixed effect, allowing us to estimate the effect of *c*_*ij*_ independently from *a*_*ij*_. Given that *b*_*ij*_ is estimated with uncertainty, and ignoring this uncertainty might inflate the differences in the rate of aging between causes of death, we also included a weight variable in each LMM, computed as $w=\frac{1}{\mathrm{var}\left(b_{\mathrm{ij}}\right)}$.

To better visualize the differences in the rate of aging between causes of death, we computed the mortality rate doubling time (MRDT):

(4) $$\text{MRDT}=\frac{\ln\left(2\right)}{b_{\mathrm{ij}}}$$

where *b*_*ij*_ is the rate of aging for country *i* and cause of death *j*. Confidence intervals (95%) of MRDT were computed as:

(5) $$\mathrm{CI}\left(\text{MRDT}\right)=\frac{\ln\left(2\right)}{b}\pm 1.96\;\left(\frac{\ln\left(2\right)}{b^{2}}\;\mathrm{SE}\;\left(b\right)\right).$$

### 2.3 Lifespan Equality for Causes of Death and Relative Contribution of Early‐Life Mortality, Adult Mortality, and Rate of Aging to Temporal Changes of Cause‐Specific Lifespan Equality

Mortality can be differently distributed across ages (more evenly or more clustered) between causes of death. We therefore computed lifespan equality as follows:

(6) e=1–G

where *G* is the Gini's coefficient of age at death computed as follows:

(7) $$G=\frac{E|X-Y|}{2\bar{x}}$$

where

(8) $$E|X-Y|=\sum_{i}\sum_{j}f_{i}f_{j}|r_{i}-r_{j}|$$

*r*_*i*_, *r*_*i*_, *f*_*i*_ and *f*_*j*_ being the age at death and the associated death frequency at ages *i* and *j*, and $\bar{x}$ being the mean age at death computed as:

(9) $$\bar{x}=\frac{\sum_{i}d_{i}r_{i}}{\sum_{i}d_{i}}$$

*d*_*i*_ being the number of deaths in age class *i*. Please note that $\bar{x}$ is not the life expectancy if people would only die from a specific cause of death, but the average age of people who died from that specific cause.

A high lifespan equality means most people die close to the same age (clustered mortality); a low lifespan equality means deaths are more spread across ages. Gini coefficients were computed using the observed number of deaths per age class for each country and cause of death, for 1955 and 2020. For the computation of both *e* and $\bar{x}$, we used the entire age range (0–85+ years). The open 85+ age class was approximated by assigning deaths to age 87.5. As with any midpoint approximation for an open‐ended interval, this truncates the upper tail of the age‐at‐death distribution, leading to a slight underestimation of mean age at death and a slight overestimation of lifespan equality (Preston et al. 2001; Kannisto 2000). However, our goal was not to provide the most accurate estimates of these parameters but instead to provide a comparative analysis between causes of death.

The contribution of different parameters describing age‐dependent mortality to temporal changes in lifespan equality for specific causes of death was assessed using a Shapley decomposition (Shorrocks 2013; Fourrey 2023). We defined the full age at death distribution as follows:

(10) $$f\left(a;p,b_{0},b_{1}\right)=\left(1-p\right)\;f_{c}\left(a\right)+p\;f_{a}\left(a;b_{0},b_{1}\right)$$

where *p* is the fraction of deaths occurring in adulthood (*a* > 30), 1 − *p* is the fraction occurring before age 30, $f_{c}\left(a\right)$ is the distribution of early‐life deaths, $f_{a}\left(a;b_{0},b_{1}\right)$ is the adult death distribution given by the Gompertz hazard model.

Over time (1955–2020), parameter values change as follows:

(11) $$\left(p_{0},b_{0,0},b_{1,0}\right)\to \left(p_{1},b_{0,1},b_{1,1}\right),$$

the distribution changes as follows:

(12) $$f_{0}\left(a\right)\to f_{1}\left(a\right),$$

and the change in lifespan equality is:

(13) $$∆e=e\left(f_{1}\right)-e\left(f_{0}\right).$$

The contribution of each parameter to Δ*e* is then:

(14) $$\varphi_{i}=\sum_{S\subseteq N\backslash \left\{i\right\}}\frac{|S|!\left(3-|S|-1\right)!}{3!}\;\left[e\left(S\cup \left\{i\right\}\right)-e\left(S\right)\right]$$

where

(15) $$N=\left\{p,b_{0},b_{1}\right\}.$$

Thus,

(16) $$∆e=\varphi_{\text{early}-\text{life}}+\varphi_{b_{0}}+\varphi_{b_{1}}$$

where $\varphi_{\text{early}-\text{life}}$ describes how much lifespan equality has changed between 1955 and 2020 due to a mortality shift from infants/children to adults, and $\varphi_{b_{0}}$ and $\varphi_{b_{1}}$ describe how much lifespan equality has changed due to changes in Gompertz parameters.

All codes were written with AI assistance and run in SAS Studio; they are provided in the online Supporting Information.

## 3 Results

### 3.1 Rate of Aging for the Main Causes of Death

**FIGURE 1:**
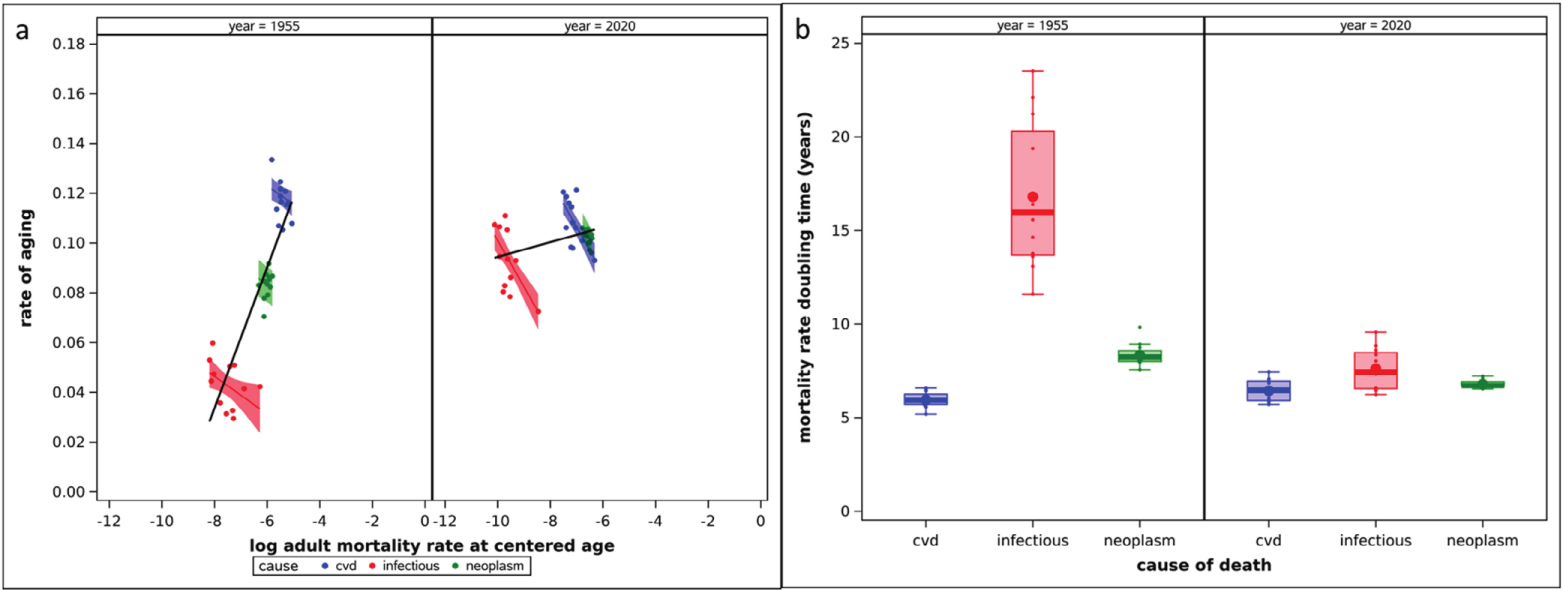
(a) Relationship between the rate of aging and the log adult mortality rate at centered age. The rate of aging and the log adult mortality rate were estimated fitting a Gompertz model to age‐specific mortality rates for specific causes of death [cardiovascular diseases (cvd), infectious and parasitic diseases and malignant neoplasm]. Each dot represents a country, the colored lines represent the fit of a LMM for each cause of death (the band representing the 95% CI), while the black line represent the across‐cause fit. *N* = 12 countries per cause of death and year. (b) Mortality rate doubling time (years) per cause of death and year. Dots represent countries; boxes represent the interquartile range (IQR); the horizontal line within each box indicates the median; the larger dot indicates the mean; whiskers extend to the most extreme values within 1.5 × IQR from the quartiles; and points beyond the whiskers indicate outliers. *N* = 12 countries per cause of death and year.

We first compared how the rate of aging varied across three major causes of death, while controlling for the other parameter of the Gompertz model (the log adult mortality rate at centered age). Using the 1955 data, as expected, the rate of aging for infectious diseases was lower than for both cardiovascular diseases and malignant neoplasm (Table 1, Figure 1a). Repeating the analysis with the 2020 data revealed the same pattern, with the rate of aging for infectious diseases remaining significantly lower than for the other two causes of death. However, whereas in 1955 the three causes of death were approximately aligned in the two‐dimensional space defined by the rate of aging and log adult mortality, this relationship became less pronounced in 2020. This change was due to the infectious diseases group shifting towards the region of lower adult mortality and higher rate of aging. In 2020, the rate of aging for cardiovascular diseases and malignant neoplasm converged to a value corresponding to approximately a 10% increase in mortality per year. A more graphical illustration of the differences in the rate of aging among causes of death is provided by the mortality rate doubling time (MRDT), which shows that MRDT has decreased for infectious and parasitic diseases between 1955 and 2020 (Figure 1b).

**TABLE 1:** Linear mixed models (LMM) investigating the effect of cause of death (infectious and parasitic diseases, cardiovascular diseases, and malignant neoplasm) on the rate of aging (the *b* parameter of the Gompertz model) while controlling for log adult mortality rate at centered age (the *a* parameter of the Gompertz model).

Year = 1955					
*Fixed effects*	*Estimate*	*95% CI*	*df*	*F*	*p*
Cause of death			2,21	100.05	< 0.0001
Cardiovascular	0.092	0.077/0.107			
Neoplasm	0.052	0.039/0.065			
Log adult mortality rate	−0.008	−0.014/−0.001	1,21	5.22	0.0329
*Random effect*	*Estimate*	*SE*	*z*	*p*	
Country	0.000022	0.000014	1.66	0.0488	

Year = 2020					
*Fixed effects*	*Estimate*	*95% CI*	*df*	*F*	*p*
Cause of death			2,21	35.98	< 0.0001
Cardiovascular	0.061	0.046/0.077			
Neoplasm	0.065	0.046/0.085			
Log adult mortality rate	−0.019	−0.025/−0.012	1,21	37.59	< 0.0001
*Random effect*	*Estimate*	*SE*	*z*	*p*	
Country	3.8e^−10^	0.000011	0.35	0.3632	

*Note:* Country identity was included in the model as a random intercept. Models were run for 1955 and 2020. We report the parameter estimates (for cause of death, infectious and parasitic diseases is the reference group) with the 95% confidence intervals, degrees of freedom, *F*, and *p* values. For the random effect, we report the parameter estimate with the standard errors, the *z*, and *p* values. *N* = 12 countries per year.

### 3.2 Rate of Aging for Causes of Death With Different Etiologies Sharing the Same Tissue/Organ Tropism

In the previous model, we compared the rate of aging between broad disease categories. Here, we restricted the comparison to causes of death with different etiologies (infectious vs. noninfectious) that share the same tissue/organ tropism. To this purpose, we compared causes of death that involve the respiratory and the digestive system. As for the previous analysis, the models were run with the 1955 and the 2020 data, except for digestive diseases for which mortality rates were too low in 2020 to extract reliable Gompertz model parameters.

**FIGURE 2:**
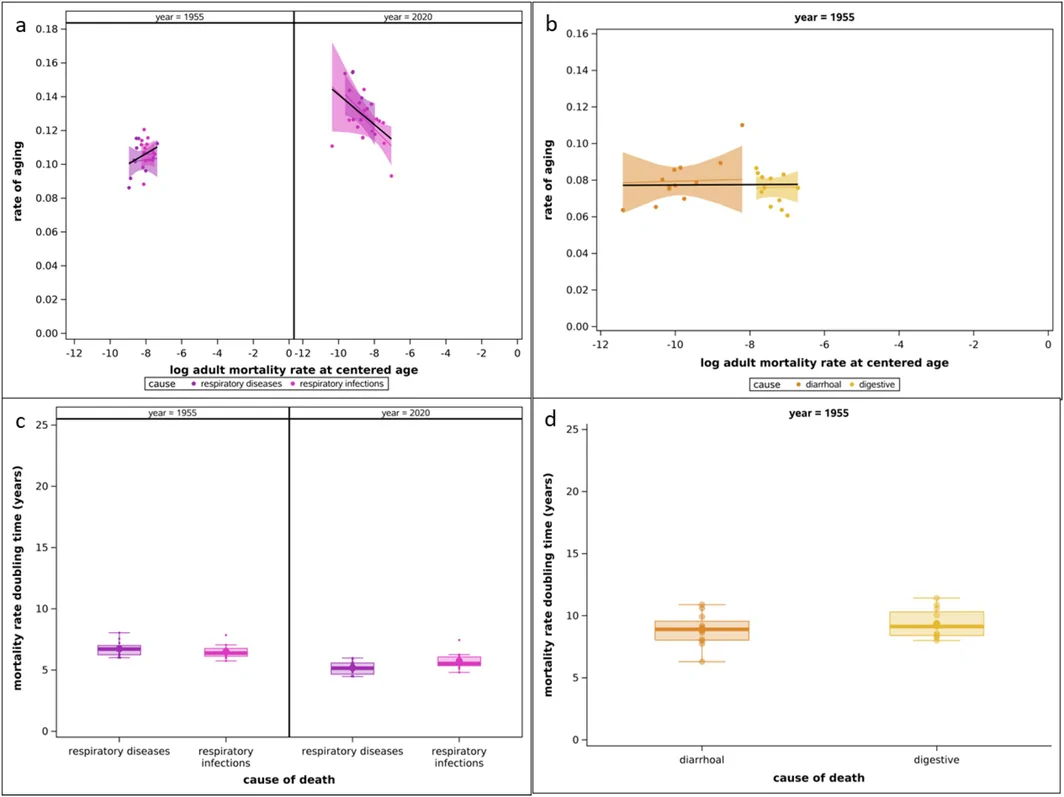
(a and b) Relationship between the rate of aging and the log adult mortality rate at centered age. The rate of aging and the log adult mortality rate were estimated fitting a Gompertz model to age‐specific mortality rates for causes of death with different etiologies (infectious and noninfectious) but similar disease tropism (a = respiratory system; b = digestive system). Each dot represents a country, the colored lines represent the fit of a LMM for each cause of death (the band representing the 95% CI), while the black line represent the across‐cause fit. (c and d) Mortality rate doubling time (years) per cause of death and year. Dots represent countries; boxes represent the interquartile range (IQR); the horizontal line within each box indicates the median; the larger dot indicates the mean; whiskers extend to the most extreme values within 1.5 × IQR from the quartiles; and points beyond the whiskers indicate outliers. *N* = 12 countries per cause of death and year.

In contrast to the previous results, we found that causes of death affecting the same tissues or organs exhibited remarkably similar rates of aging, regardless of baseline adult mortality levels or disease etiology. This pattern held for infectious versus noninfectious respiratory diseases in both years, and for infectious versus noninfectious digestive diseases in 1955 (Table 2, Figure 2a,b). Additionally, calculating the MRDT further illustrated the stability of the rate of aging in these pairwise comparisons over time (Figure 2c,d).

**TABLE 2:** Linear mixed models (LMM) investigating the effect of etiology (infectious vs. noninfectious) for diseases with similar tropism (respiratory system and digestive system) on the rate of aging (the *b* parameter of the Gompertz model) while controlling for log adult mortality rate at centered age (the *a* parameter of the Gompertz model).

Respiratory system year = 1955					
*Fixed effects*	*Estimate*	*95% CI*	*df*	*F*	*p*
Etiology			1,10	0.95	0.3524
Noninfectious	−0.003	−0.010/0.004			
Log adult mortality rate	0.001	−0.010/0.012	1,10	0.08	0.7838
*Random effect*	*Estimate*	*SE*	*z*	*p*	
Country	0.000038	0.000029	1.30	0.0971	

Respiratory system year = 2020					
*Fixed effects*	*Estimate*	*95% CI*	*df*	*F*	*p*
Etiology			1,10	0.27	0.6124
Noninfectious	0.003	−0.010/0.016			
Log adult mortality rate	−0.011	−0.021/−0.0001	1,10	5.10	0.0475
*Random effect*	*Estimate*	*SE*	*z*	*p*	
Country	0.000062	0.000049	1.26	0.1046	

Digestive system year = 1955					
*Fixed effects*	*Estimate*	*95% CI*	*df*	*F*	*p*
Etiology			1,10	0.18	0.6787
Noninfectious	−0.005	−0.031/0.021			
Log adult mortality rate	0.001	−0.009/0.010	1,10	0.02	0.8959
*Random effect*	*Estimate*	*SE*	*z*	*p*	
Country	0.000018	0.000022	0.82	0.2074	

*Note:* Country identity was included in the model as a random intercept. We ran three models (for diseases with digestive tropism the model was only run for 1955). We report the parameter estimates (for etiology, infectious is the reference group) with the 95% confidence intervals, degrees of freedom, *F* and *p* values. For the random effect, we report the parameter estimate with the standard errors, the *z* and *p* values. *N* = 12 countries per tropism and year.

**FIGURE 3:**
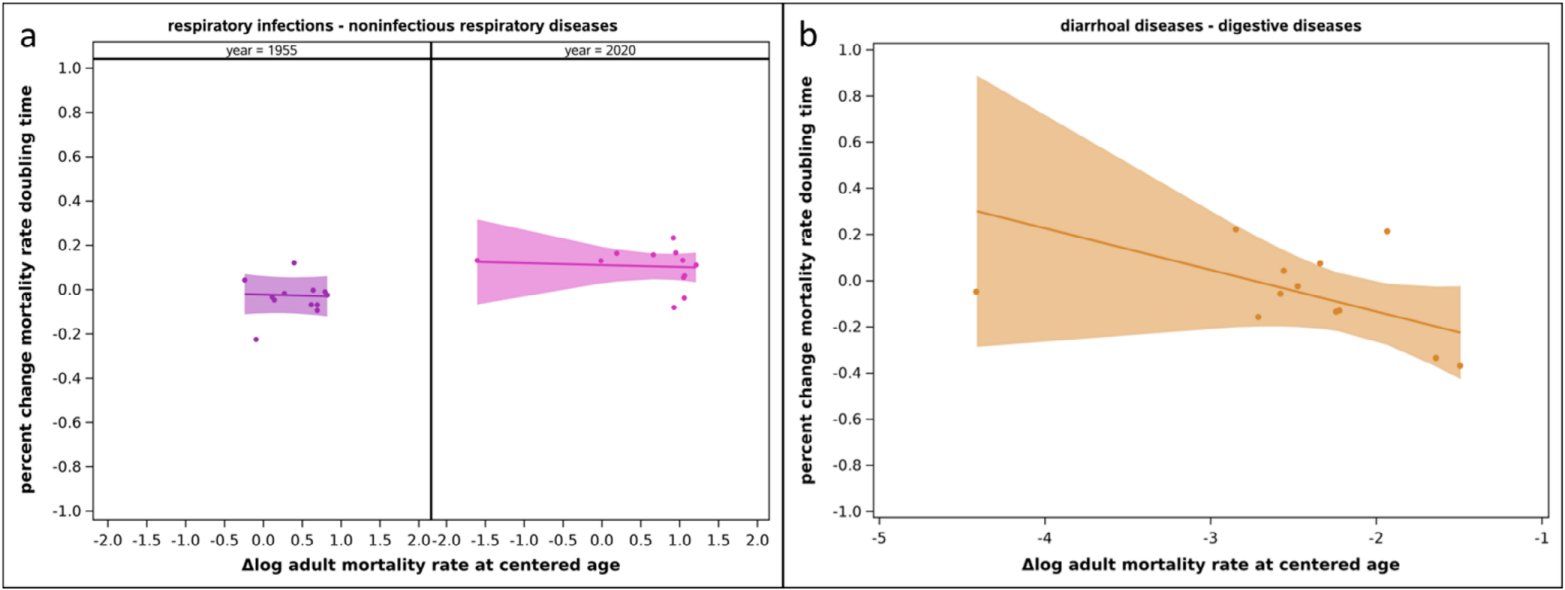
(a) Relationship between percent changes in mortality rate doubling time and Δlog adult mortality rate at centered age computed between causes of death targeting the respiratory system having different etiologies (respiratory infections minus noninfectious respiratory diseases) in 1955 and 2020. (b) Relationship between percent changes in mortality rate doubling time and Δlog adult mortality rate at centered age computed between causes of death targeting the digestive system having different etiologies (diarrhoal diseases minus noninfectious digestive diseases) in 1955. Each dot represents a country, the lines represent the fit of a LMM for each pairwise comparison (the band representing the 95% CI). *N* = 12 countries per pairwise comparison and year.

To further assess the magnitude of differences in aging rates for specific causes of death, we calculated the percent change in MRDT (∆MRDT) for pairwise comparisons of causes of death with similar tropism (infectious minus noninfectious) and regressed this against the difference in log adult mortality rate at centered age. The models indicated that differences in the rate of aging were essentially independent of differences in log adult mortality for both the respiratory and digestive system (Table 3, Figure 3). Furthermore, while cause‐specific mortality levels varied substantially across countries, the relative differences in MRDT were modest, generally within ±20% (corresponding to a 1‐ to 2‐year difference in MRDT). Overall, this confirms that causes of death with similar organ specificity differ primarily in mortality levels rather than in the rate of aging.

**TABLE 3:** Linear mixed models (LMM) investigating the effect of Δlog adult mortality rate at centered age on the percent change in MRDT.

Respiratory system					
*Fixed effects*	*Estimate*	*95% CI*	*df*	*F*	*p*
Δlog adult mortality rate	−0.009	−0.082/0.064	1,10	0.06	0.8048
Year			1,10	12.85	0.0050
2020	0.134	0.051/0.216			
*Random effect*	*Estimate*	*SE*	*z*	*p*	
Country	0.005	0.003	1.67	0.0479	

Digestive system					
*Fixed effects*	*Estimate*	*95% CI*	*df*	*F*	*p*
Δlog adult mortality rate	−0.180	−0.431/0.071	1,10	2.56	0.1404

*Note:* Percent changes in MRDT and Δlog adult mortality rate were computed between infectious and noninfectious causes of death per system (respiratory system and digestive system) and year (1955 and 2020 for the respiratory system, and 1955 for the digestive system). Country identity was included in the model as a random intercept. We report the parameter estimates (for year, 1955 is the reference group) with the 95% confidence intervals, degrees of freedom, *F* and *p* values. For the random effect, we report the parameter estimate with the standard errors, the *z* and *p* values. *N* = 12 countries per system.

### 3.3 Lifespan Equality for Causes of Death and Relative Contribution of Early‐Life Mortality, Adult Mortality and Rate of Aging to Temporal Changes of Cause‐Specific Lifespan Equality

**FIGURE 4:**
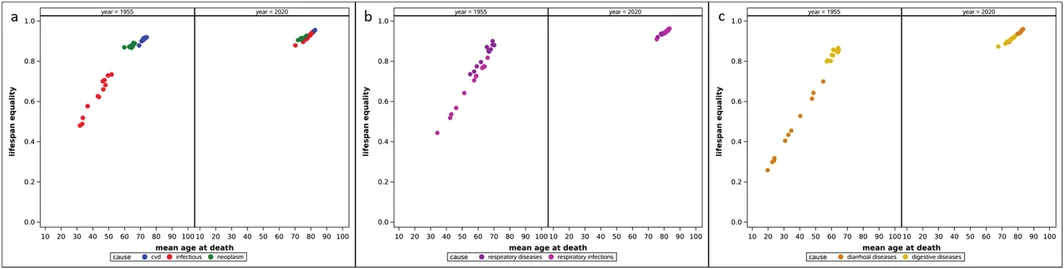
Relationship between lifespan equality and mean age at death for (a) broad categories of causes of death [cardiovascular diseases (cvd), infectious and parasitic diseases, malignant neoplasm], (b) causes of death with different etiologies (infectious, noninfectious) but sharing the same tropism for the respiratory system, (c) causes of death with different etiologies (infectious, noninfectious) but sharing the same tropism for the digestive system. Each dot represents one country. Dots represent countries; boxes represent the interquartile range (IQR); the horizontal line within each box indicates the median; the larger dot indicates the mean; whiskers extend to the most extreme values within 1.5 × IQR from the quartiles; and points beyond the whiskers indicate outliers. *N* = 12 countries per cause of death.

We first analyzed lifespan equalities for the broad categories of causes of death (infectious and parasitic diseases, cardiovascular diseases and malignant neoplasm). Lifespan equalities linearly increased as mean age at death increased, implying that when people die at older ages for a given cause, they also have less variability in the age at death (Figure 4a). For the 1955 data, infectious diseases clustered in a distinct area of the two‐dimensional space defined by lifespan equality and mean age at death (lower values) compared to the other causes of death, showing that deaths from infectious diseases occurred across a broader range of ages (infants, children, young adults, older adults). For both cardiovascular diseases and malignant neoplasm, values grouped in the upper‐right corner showing high clustered mortality around high mean age at death (Figure 4a). In 2020, the lifespan equality moved upward/rightward for the three causes of death, reflecting the epidemiological transition where deaths are increasingly determined by the aging process, even for infectious causes (Figure 4a). Exactly the same pattern was found when focusing on pairwise comparisons between causes of death (infectious vs. noninfectious) with similar organ specificity: lifespan equalities for infectious causes moved upwards/rightwards, indicating more clustered mortality at old ages (Figure 4b,c).

**FIGURE 5:**
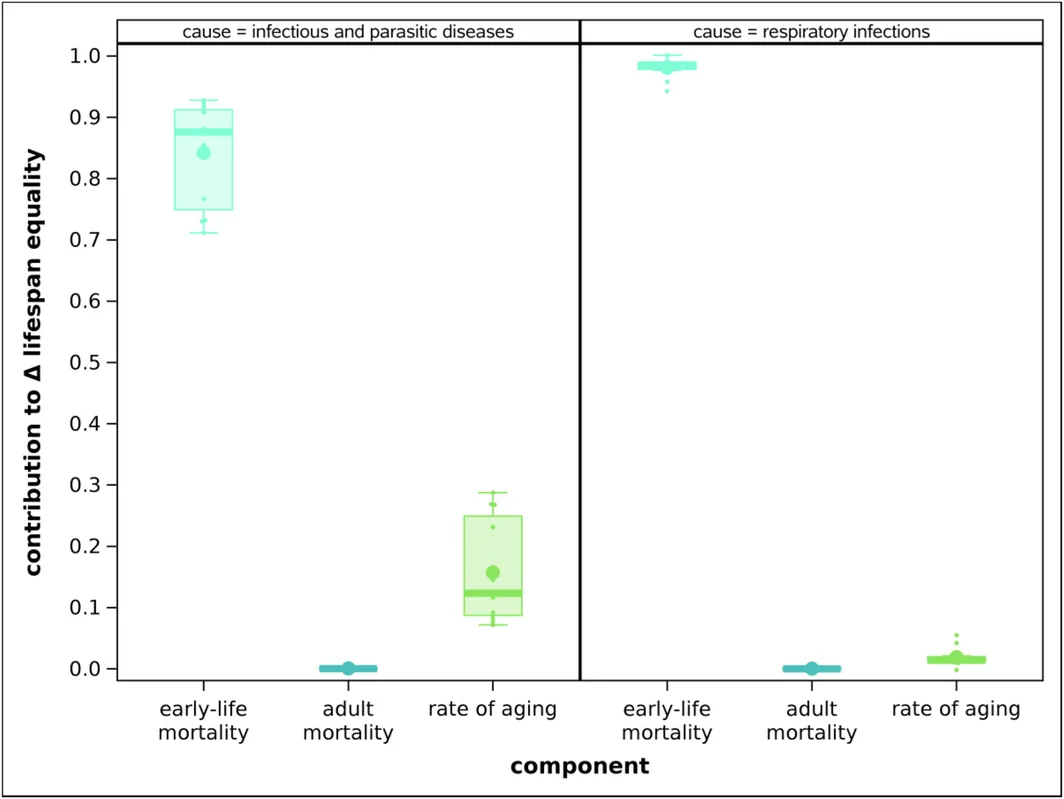
Contribution of changes in early‐life mortality, adult mortality and rate of aging to the Δ lifespan equality (between 2020 and 1955) for infectious and parasitic diseases as a whole and for respiratory infections. Dots represent countries; boxes represent the interquartile range (IQR); the horizontal line within each box indicates the median; the larger dot indicates the mean; whiskers extend to the most extreme values within 1.5 × IQR from the quartiles; and points beyond the whiskers indicate outliers. *N* = 12 countries per cause of death.

Temporal changes in lifespan equality can result from varying contributions of parameters describing the distribution of mortality across ages. To investigate this, we quantified the contributions of changes in early‐life mortality, adult mortality, and the rate of aging to temporal changes in lifespan equality for infectious diseases (broad category) and respiratory infections, using a Shapley decomposition. We focused on these two causes of death because they exhibited large changes in lifespan equality. Temporal changes in lifespan equality were predominantly driven by shifts in mortality from early‐life to adulthood, with changes in the rate of aging contributing minimally (Figure 5). For infectious and parasitic diseases as a broad category, although changes in early‐life mortality accounted for most of the change in lifespan equality, changes in the rate of aging still contributed about 17% (Figure 5a). This finding supports the earlier analysis showing that the rate of aging has increased over time for infectious and parasitic diseases. In contrast, for respiratory infections, changes in the rate of aging contributed almost nothing to the change in lifespan equality, with nearly 100% of the temporal change attributable to changes in early‐life mortality (Figure 5b).

## 4 Discussion

Historically, infectious diseases have represented a major mortality source in human societies, with both considerable demographic effects and genomic signatures (Dobson and Carper 1996; Karlsson et al. 2014; Izdebski et al. 2022). Infectious diseases have traditionally been considered as an extrinsic source of mortality depending on the exposure to transmissible microorganisms. However, the outcome of the infection also depends on intrinsic factors (how well the immune response deals with the infection and how well the repair mechanisms restore homeostasis). In a previous work (Sorci and Faivre 2022), it was shown that the untreated case fatality rate (CFR, the proportion of dead among untreated infected individuals) for several human infectious diseases varies with age according to a typical J function, where CFR is higher at both extremes of the age range (infant and elderly). Although being the dominant pattern, in some cases CFR monotonically increased or decreased with age, possibly reflecting specific processes such as lifetime persistent immunity. However, while assessing the rate of increase of CFR with age allows one to infer the age‐dependent vulnerability, it does not allow one to infer the demographic impact of infectious diseases, since only the infected population is considered. Here, we analyzed how fast mortality due to infectious diseases increases with age in the general population, and compared it to causes of death more strictly associated to intrinsic factors. By computing the parameters of the Gompertz model, we showed that the rate at which mortality increases with age (over the 30–85 years range) is lower for infectious disease compared to cardiovascular diseases and malignant neoplasm. This result refers to 1955, in a period where the epidemiological transition was likely not fully completed and where mortality from infectious diseases was hitting a relatively large range of ages. When repeating the analysis using more contemporary data (2020), we found that the rate of aging for infectious diseases converged towards the values of cardiovascular diseases and neoplasm. This result shows that while in the mid‐20th century, infectious diseases mortality still had a large age‐independent component, nowadays infectious diseases mortality is predominantly age‐dependent, at least in the high‐income countries considered here. The Gompertz model has been widely used in demographic research to compare the age‐dependent increase in all‐causes mortality across populations, countries, and calendar periods (Avraam et al. 2014; Finch et al. 2014; Cohen et al. 2018; Tai and Noymer 2018). However, studies involving specific causes of death are much less numerous. For instance, Goldstein and Lee (2020) and Sasson (2021) analyzed mortality due to COVID‐19 and found that COVID‐19 mortality increased with age at the same rate as all‐causes mortality (ca. 10% per year). Sasson (2021) also compared MRDT of COVID‐19 to other infectious diseases of the lower respiratory tract and found that mortality rate increased with age at a similar pace, irrespective of the causative infectious agent.

The burden of mortality due to infectious diseases has globally decreased over the last century, albeit the pace and the magnitude of the effect are more pronounced in high‐income compared to low‐income countries, due to improved sanitation/hygiene, more effective antimicrobial treatments, and better health care systems (Armstrong et al. 1999; Birchenall 2007; GBD 2019 Diseases and Injuries Collaborators 2020). Here, we show that while the overall mortality burden has decreased, the age‐dependent schedule has shifted from early to late mortality over time, resulting in a steeper, age‐dependent increase in mortality due to infectious diseases in contemporary time. This result does not follow the general trend for all‐causes mortality, where the rate of aging has been shown to be relatively constant over time and the onset of aging being delayed (Patricio 2026). The temporal increase in the rate of aging for infectious diseases raises an interesting question. Why did improvements in sanitation and antimicrobial treatments (including vaccination) not lead to a similar reduction in mortality rates among people of all ages? Aging is associated with a series of alterations of homeostasis, ultimately compromising vital functions (López‐Otín et al. 2023). During an infection, once the pathogen has successfully established within the host, the outcome of the interaction essentially depends on the host's capacity to limit pathogen proliferation and on the capacity to repair the damage caused by the infection (which potentially includes also the damage induced by the immune response) (Schneider and Ayres 2008). Generally speaking, improved sanitation/hygiene or vector control aim at reducing the exposure rate, while antimicrobial treatments act as a surrogate of the immune system by either killing the pathogen or by stopping its replication. The efficacy of sanitation and vector‐control measures is expected to be largely age‐independent because these interventions primarily reduce environmental exposure rather than host susceptibility; for example, vector control should similarly reduce the exposure to pathogens carried by the vectors for young and old hosts. On the contrary, the clinical efficacy of antimicrobial treatments is not identical for young and old hosts. The effect of age on clinical efficacy depends on several factors such as the pharmacokinetics and pharmacodynamics of the drug, and more importantly the inherent contribution of the host immune response to clear the infection (Ramsden et al. 2008; Nikolich‐Zugich 2018; Wong et al. 2020; Palacios‐Pedrero et al. 2021; Fu et al. 2024). In addition to this age‐dependent effect on resistance, aging is also associated with a reduced capacity to restore/repair damage caused by infections (Sorci et al. 2021; Sanchez et al. 2026). This suggests that despite the improvement in treatments and health care systems, the extension of mean age at death for infectious diseases should reside in the postponement of the possible biological limits set by the host's capacity to clear the infection and/or to regenerate and repair the damage induced by the infection. The idea that tolerance to infection is an important defense strategy has been brought in the last years in the evolutionary ecology literature (Read et al. 2008; Råberg et al. 2009), but disruption of tolerance is also a major determinant of disease severity in human infectious diseases (Regoes et al. 2014; Mankowski et al. 2020).

Different organs and tissues differ in their capacity to withstand an insult (infectious and noninfectious), depending on their repair/renewal potential (Medzhitov et al. 2012). For instance, epithelia generally have high regenerative capacity while neurons or cardiomyocytes have no or extremely low proliferative capacity. As a consequence, damage incurred by the brain or the heart has potentially more severe consequences compared to organs with higher repair capacity. Comparative work has supported the importance of tissue tropism in shaping the severity of infectious diseases (Brierley et al. 2019; McCall 2021). Infectious and noninfectious diseases with similar organ specificity (i.e., targeting the same organs) can converge to similar physiopathologies, usually (but not exclusively) involving the immune response. For instance, the phenotype of liver injury caused by viral infection (e.g., hepatitis) or drug adsorption (e.g., drug‐induced liver injury) is largely dominated by immune‐mediated hepatocyte cytotoxicity (Stravitz and Lee 2019). Similarly, the pathophysiology of infectious and secretory/toxic diarrhea involve cAMP‐mediated chloride secretion with massive water efflux into the intestinal lumen (Field 2003; Harris et al. 2012). Broadly speaking, these shared mechanisms can include immune‐mediated injury, mitochondrial dysfunction, oxidative stress, membrane destabilization, and dysregulation of ion transport and cellular homeostasis (Sies and Jones 2020; Medzhitov 2021; Szabo and Szewczyk 2023; Zong et al. 2024). Therefore, different triggers can produce similar pathological phenotypes. In agreement with the idea of the importance of organ specific defense (e.g., tolerance) mechanisms, we found that the rate of aging was strikingly similar when the comparison between causes of death was restricted to diseases hitting the same tissues/organs. In other words, when considering infectious and noninfectious diseases with similar organ specificity, the mortality increased with age at a constant rate, both when using the 1955 data and the more contemporary 2020 data. Importantly, rates of aging were similar between infectious and noninfectious causes of death, despite fairly large differences in adult mortality rate, between causes and years. Thus, shared physiopathology mechanisms of diseases hitting the same systems might play a role in driving the age‐specific increase in mortality rate.

The increase in life expectancy at birth goes in parallel with a reduction of its variance. In other words, as life expectancy increases, deaths tend to cluster around a narrower range of ages (Colchero et al. 2016; Aburto et al. 2020). This general pattern has been reported across a large range of human populations covering different mortality regimes (Vaupel et al. 2021). To the best of our knowledge, our work is the first to adopt this framework, showing that for every cause of death, higher mean age at death (i.e., deaths occurring later in life) is strongly associated with higher lifespan equality (i.e., deaths are concentrated within a narrower age range). While previous studies have analyzed this relationship for all‐causes mortality (Colchero et al. 2016; Aburto et al. 2020), or have estimated the contribution of causes of death to the all‐causes lifespan equality (Permanyer and Vigezzi 2024), we show here that the same pattern exists within specific causes of death, suggesting a universal demographic regularity: as the modal age of death increases, variation in age at death decreases. This reveals that even within cause‐specific mortality, aging processes lead to a compression of mortality risk around a typical age of death.

The analysis of lifespan equality for causes of death was consistent with the results of the comparison of the rate of aging. In 1955, lifespan equality for infectious diseases ranged from 0.48 to 0.77. As a comparison, in 1955, lifespan equalities for cardiovascular diseases and malignant neoplasm were homogenously around 0.9 across countries. However, by 2020, lifespan equality (and mean age at death) for infectious diseases fully converged towards the values of cardiovascular diseases and malignant neoplasm. This therefore shows that the contemporary mortality regime of infectious diseases is dominated by age‐dependent factors and that intrinsic causes may now play a larger role in mortality due to infectious diseases than extrinsic components. Another interesting pattern that emerges from the analysis of the temporal changes in the relationship between lifespan equality and mean age at death for specific causes of death is that, even within a relatively homogenous sample of high‐income countries, the epidemiological transition occurred earlier for some countries. This is clearly shown by the scatter of the lifespan equalities across countries in 1955. This between‐country variability completely vanished by 2020, suggesting that all countries had completed the epidemiological transition. The same general trend was also observed when focusing on causes of death involving diseases with similar organ specificity but different etiologies, with mortality becoming more clustered at old ages over time and particularly so for diseases with an infectious etiology.

Temporal changes in lifespan equality can be due to different contributions of the parameters describing the distribution of mortality across ages. Using a Shapley decomposition, we showed that by far the largest contribution to the Δ lifespan equality comes from changes in early‐life mortality, with virtually nil contributions from adult mortality and the rate of aging. These results parallel those reported by Colchero et al. (2021) on all‐causes mortality in human and nonhuman primate populations.

### 4.1 Implications for Further Extension of Healthspan

High‐income countries have completed their epidemiological transition and infectious diseases are now senescent diseases. The spectacular gain in terms of life expectancy over the last decades has been achieved from shifting mortality schedule from early‐ to late‐life (Bergeron‐Boucher et al. 2015; Zuo et al. 2018; Vaupel et al. 2021; Haas and Sunde 2025). Further extension should now focus on flattening the rate at which mortality increases with age and reducing mortality compression at old age (Patricio 2026). Whether this goal can be ever attained and the magnitude of the life expectancy extension will depend on whether the current mortality schedule has hit the limit inherent to the capacity of tissues and organs to repair age‐associated damage. With this respect, we suggest that public health interventions should focus on the preservation and restoration of functions in the elderly and treatments should aim at improving the capacity to regenerate damaged tissues. Although this is a tantalizing task, such interventions have already been implemented to treat degenerative diseases of the elderly, albeit they are not explicitly formalized in the framework of the dynamical relationship between damage and repair. Table 4 reports some selected examples of interventions that aim at restoring functions, both as preventive strategies (before health is compromised) or as treatments.

**TABLE 4:** Tolerance‐enhancing interventions, including preventive strategies, in the elderly.

Intervention category	Representative interventions	Primary mechanisms	Effect on tolerance	Vulnerabilities in the elderly	References
Nutritional optimization	Adequate protein intake; micronutrient repletion (vitamin D, B12, iron)	Support of mitochondrial function; maintenance of protein synthesis and immune competence	Increased metabolic reserve and resilience to infection	High prevalence of malnutrition and sarcopenia in elderly	Volkert et al. (2019), Singer et al. (2019)
Physical activity and frailty prevention	Resistance training; aerobic exercise; fall prevention programs	Prevention of muscle catabolism; preservation of neuromuscular function	Improvement of global physiological resilience	Frailty strongly predicts adverse outcomes during infection	Clegg et al. (2013), Schweickert et al. (2009)
Microbiome resilience and restoration	Diet promoting microbiota diversity and preventing dysbiosis; avoidance of unnecessary antibiotics; fecal microbiota transplant	Maintenance and restoration of gut microbial diversity; preservation of mucosal barrier function	Improved resilience towards intestinal infection	Increased risk of dysbiosis and recurrent infections	Buffie and Pamer (2013), van Nood et al. (2013), Blaser (2016), Fu et al. (2025)
Anti‐inflammatory modulation	Regular physical activity; anti‐inflammatory treatments	Reduction of chronic low‐grade inflammation; attenuation of excessive inflammatory response	Reduction of damage due to an overreacting response	Dysregulated inflammatory response (inflammaging)	Annane et al. (2018), Furman et al. (2019), RECOVERY Collaborative Group (2021)

In 2024 the proportion of people over 65 years represented about 20% of the overall population in high‐income countries (more than three times higher than the proportion of children under 5 years) (World Bank 2025) and the proportion of elderly is projected to reach 25%–27% of the population in 2050 (United Nations Population Division 2022). In high‐income countries, population aging is also projected to increase public health expenditure by approximately 0.5% to 1% of GDP by 2050, while long‐term care spending, more directly driven by aging, is expected to rise from around 1.5% to up to 3%–4% of GDP (Organisation for Economic Co‐operation and Development 2024–2025). In this context, preventing age‐associated damage (Gladyshev et al. 2021) and implementing treatments aiming at repairing damage and restoring homeostasis represent the main avenues to enhance healthspan (Goldberg and Dixit 2015; Dugan et al. 2023).

Advanced age is also characterized by the occurrence of several pathologies sharing environmental and socio‐economic determinants. The interaction between environmental, socio‐economic factors and the incidence of multimorbidities has been named syndemics (Singer et al. 2017). Although the concept of syndemics is not specific to older adults, advanced age is associated with increased frailty and multimorbidity, making elderly populations particularly susceptible to synergistic interactions between socially determined vulnerabilities and cooccurring diseases (Singer et al. 2017). The recent SARS‐CoV‐2 outbreak has provided a striking example of syndemics, where COVID‐19 mortality was particularly severe in deprived populations suffering from other socially determined diseases (McGowan and Bambra 2022; Sorci 2024). Therefore, public health measures should also focus on the vulnerability of the elderly to the socio‐economic triggers of disease.

### 4.2 Limitations

We would like to acknowledge a few limitations of this work. We only analyzed a sample of 12 high‐income countries at two time periods; therefore, the results only apply to these specific conditions. In particular, they might not apply when considering a broader sample of middle‐ and low‐income countries with different mortality regimes.

We used broad categories to attribute causes of death, by grouping all infectious diseases together or all cancers together. Of course, this merges diseases with very different incidences or pathophysiologies in the same categories. This choice was motivated by the need to have reliable estimates of mortality rates based on a large number of deaths and consistent attributions between different ICD versions, but we acknowledge that this certainly mixes different mortality regimes within each category. For the same reason, we also analyzed mortality rates for both sexes combined. Women and men may be susceptible to different causes of death, which could affect the rate at which mortality increases with age. Further work in this area should attempt to estimate sex‐specific differences in cause‐specific mortality rates.

The analysis of disease tropism was conducted considering only two systems, the respiratory and the digestive system. Physiological systems differ in their vulnerability to damage and their tissue repair potential (Medzhitov et al. 2012). Therefore, the finding of a similar rate of aging for causes of death with similar tissue/organ specificity might only apply to the systems considered here. The reliability of the findings also rests on the quality of the data, in particular their completeness and the accuracy of the attribution of the causes of death. This might be an issue for early data (1955) and for the oldest age classes where, as mentioned above, death might arise due to the interactive effects of cooccurring pathologies. However, this should be less of an issue for the 2020 data, as the causes of death should be accurately attributed and the data should be 100% complete.

Finally, we acknowledge that analyzing temporal changes in the rate of aging incorporates both period and cohort effects (Gavrilov and Gavrilova 2023). The objective of the present study, however, was to compare the rate of aging for specific causes of death rather than decomposing temporal trends into period and cohort components. Such a decomposition would necessitate a different modelling framework and additional assumptions due to the age‐period‐cohort identification problem (Carstensen 2007), which was beyond the scope of this analysis.

## 5 Conclusion

We adopted the approach usually implemented in evolutionary demography to compare age‐dependent mortality across species, or over space and time within species (Lemaitre et al. 2020), to the particular cases of mortality attributed to specific causes. This allowed us to uncover a series of interesting trends. First, we found that mortality caused by infectious diseases, considered as a broad cause of death, is now largely dominated by its intrinsic component (in high‐income countries). In other words, contemporary mortality due to infectious diseases follows the same schedule as degenerative diseases like cardiovascular pathologies or cancers. Second, we found that the rate at which mortality increases with age (from adulthood onwards), the rate of aging, is remarkably similar for causes of death with similar organ specificity, independent from their etiology (infectious or noninfectious). These results suggest that tissue/organ tolerance mechanisms (damage repair) might play a role in this invariant rate of aging.

## Funding

The authors have nothing to report.

## Conflicts of Interest

The authors declare no conflicts of interest.

## Supporting information


**Table S1:** List of high‐income countries, causes of death (with the disease etiology and tropism), and years (with the ICD version) included in the analysis.
**Table S2:** Variance—covariance matrix for the parameters [*a* = log adult mortality rate at centered age (i.e., 57.5 years); *b* = the slope of the relationship between log adult mortality rate and age] of Gompertz models ran over the 30–85 years age range (one model per country). For illustrative purposes, we only report the matrix referring to the infectious and parasitic diseases cause of death for 1955.
**Figure S1:** Fit of the Gompertz model per country and cause of death (broad categories) for 1955 and 2020.
**Figure S2:** Fit of the Gompertz model per country and cause of death (diseases with an infectious or noninfectious etiology but with similar organ specificity) for 1955 and 2020.

## Data Availability

The data that support the findings of this study come from a public domain and are available at https://www.who.int/data/data‐collection‐tools/who‐mortality‐database.
