# Supplementary material for: A Comparative Analysis of Aging Across Causes of Death
Source: Evol Appl. 2026 Sep 1;19(9):e70316. doi: 10.1111/eva.70316 (PMC13534842; doi:10.1111/eva.70316)
Supplement: Supplementary file 1 [file EVA-19-e70316-s001.docx]

**Online supplementary material**

**A comparative analysis of aging across causes of death**

Table S1. List of high-income countries, causes of death (with the disease etiology and tropism), and years (with the ICD version) included in the analysis.

| **Country** |  |  |
| --- | --- | --- |
| Australia |  |  |
| Austria |  |  |
| Belgium |  |  |
| Canada |  |  |
| France |  |  |
| Italy |  |  |
| Japan |  |  |
| Netherlands |  |  |
| Spain |  |  |
| Switzerland |  |  |
| UK |  |  |
| USA |  |  |
| **Causes of death** | **Disease etiology** | **Tropism** |
| cardiovascular diseases | noninfectious |  |
| diarrhoal diseases | infectious | digestive system |
| digestive diseases | noninfectious | digestive system |
| infectious and parasitic diseases | infectious |  |
| malignant neoplasm | noninfectious |  |
| respiratory diseases | noninfectious | respiratory system |
| respiratory infections | infectious | respiratory system |
| **Year** | **ICD version** |  |
| 1955 | ICD-7 |  |
| 2020 | ICD-10 |  |

Table S2. Variance – covariance matrix for the parameters [a = log adult mortality rate at centered age (i.e., 57.5 years); b = the slope of the relationship between log adult mortality rate and age] of Gompertz models ran over the 30 - 85 years age range (one model per country). For illustrative purposes, we only report the matrix referring to the infectious and parasitic diseases cause of death for 1955.

| **Australia** |  | *a* | *b* |
| --- | --- | --- | --- |
|  | *a* | 0.001354 | 3.82E-12 |
|  | *b* |  | 5.41E-06 |
| **Austria** |  | *a* | *b* |
|  | *a* | 0.001556 | 3.55E-12 |
|  | *b* |  | 6.22E-06 |
| **Belgium** |  | *a* | *b* |
|  | *a* | 0.002699 | 3.59E-12 |
|  | *b* |  | 1.08E-05 |
| **Canada** |  | *a* | *b* |
|  | *a* | 0.001187 | 3.89E-12 |
|  | *b* |  | 4.75E-06 |
| **France** |  | *a* | *b* |
|  | *a* | 0.000645 | 3.48E-12 |
|  | *b* |  | 2.58E-06 |
| **Italy** |  | *a* | *b* |
|  | *a* | 0.000621 | 3.51E-12 |
|  | *b* |  | 2.48E-06 |
| **Japan** |  | *a* | *b* |
|  | *a* | 0.002038 | 3.08E-12 |
|  | *b* |  | 8.15E-06 |
| **Netherlands** | | *a* | *b* |
|  | *a* | 0.001603 | 3.81E-12 |
|  | *b* |  | 6.41E-06 |
| **Spain** |  | *a* | *b* |
|  | *a* | 0.00117 | 3.32E-12 |
|  | *b* |  | 4.68E-06 |
| **Switzerland** | | *a* | *b* |
|  | *a* | 0.000455 | 3.57E-12 |
|  | *b* |  | 1.82E-06 |
| **UK** |  | *a* | *b* |
|  | *a* | 0.001439 | 3.72E-12 |
|  | *b* |  | 5.76E-06 |
| **USA** |  | *a* | *b* |
|  | *a* | 0.00039 | 3.83E-12 |
|  | *b* |  | 1.56E-06 |

Figure S1. Fit of the Gompertz model per country and cause of death (broad categories) for 1955 and 2020.

**1955**

**2020**

Figure S2. Fit of the Gompertz model per country and cause of death (diseases with an infectious or noninfectious etiology but with similar organ specificity) for 1955 and 2020.

**1955	2020**

**Codes used to run the Gompertz models, the LMMs, to compute mean age at death and lifespan equality, and the Shapley decomposition**

***/* code to run the Gompertz models (one example is provided referring the respiratory infections in 1955 */***

data a;

set mortality1955;

if cause="respiratory infections"; /* selecting one cause of death */

if age >30 and age < 85; /* selecting the 30 – 85 age classes */

agec=age-57.5; /* centering age */

m=death_rate/100000; /* transforming death rate x100,000 to mortality rates */

logm = log(m); /* log-transforming mortality rates */

proc sort;

by country ;

run;

ods output ParameterEstimates=params;

ods output CovMatParmEst=cov;

ods trace on;

proc nlmixed data=a cov;

parms

a = -8	/* initial parameter values */

b	= 0.11

log_sres = -1;

sres = exp(log_sres);

/* Gompertz hazard function with reference age = 57.5 */

eta = exp(a + b * (agec));

/* Log of predicted rate */

log_eta = log(eta);

/* Model for log death rates */

model logm ~ normal(log_eta, sres*sres);

predict log_eta out=eta;

by country ;

run;

ods output close;

proc sort;

by age pred;

run;

ods listing gpath="/home/gabriele.sorci/" dpi=600; /* code to draw the graph with the Gompertz fit */

ods graphics / reset

imagename="fig"

outputfmt=png

width=4in

height=4in;

ods graphics / reset

imagename="fig"

outputfmt=png

width=4in

height=4in;

title "respiratory infections" ;

proc sgpanel noautolegend;

styleattrs

datasymbols=(CircleFilled)

datacontrastcolors=(bippk )

datacolors=(bippk );

panelby country / columns=3 rows=4;

scatter x=age y=logm / group=country

markerattrs=(size=5px)

jitter

jitterwidth=0.5;

series x=age y=Pred/group=country;

band x=age upper=Upper lower=Lower/group=country fill transparency=0.5;

rowaxis

label="log mortality rate"

values=(-14 to 0 by 2)

gridattrs=(color=white)

labelattrs=(color=black family=Arial size=11 weight=bold)

valueattrs=(color=black family=Arial size=10);

colaxis

label="age (yrs)"

values=(0 to 90 by 10)

gridattrs=(color=white)

labelattrs=(color=black family=Arial size=11 weight=bold)

valueattrs=(color=black family=Arial size=10);

run;

quit;

***/* code to run the LMM on Gompertz parameters (one example is provided referring to the comparison between infectious and parasitic diseases, cardiovascular diseases and malignant neoplasm) */***

data a;

set gompertz;

w = 1 / (se_b*se_b); /* computing weights */

if cause = 'cvd' or cause = 'neoplasm' or cause = 'infectious'; /* selecting the causes of death to compare */

proc mixed data=a covtest;

class country cause(ref='infectious') ;

weight w;

model b = a cause / solution cl outpm=pred;

random intercept / subject=country;

lsmeans cause/diff=all adjust=bon;

by year;

run;

proc sort ;

by cause a pred;

run;

ods listing gpath="/home/gabriele.sorci/" dpi=600; /* code to draw the graph */

ods graphics / reset

imagename="figure"

outputfmt=png

width=8in

height=6in;

ods graphics / reset

imagename="figure"

outputfmt=png

width=8in

height=6in;

title ;

proc sgpanel data=pred ;

styleattrs

datasymbols=(CircleFilled)

datacontrastcolors=(blue red green)

datacolors=(blue red green);

panelby year / columns=2 rows=1;

scatter x=a y=b / group=cause

markerattrs=(size=5px)

jitter

jitterwidth=0.5;

series x=a y=Pred/group=cause lineattrs=(pattern=solid) ;

band x=a upper=Upper lower=Lower/group=cause fill transparency=0.5;

reg x=a y=b/ lineattrs=(color=black) nomarkers;

rowaxis

label="rate of aging"

values=(0 to 0.18 by 0.02)

gridattrs=(color=white)

labelattrs=(color=black family=Arial size=11 weight=bold)

valueattrs=(color=black family=Arial size=10);

colaxis

label="log adult mortality rate at centered age"

values=(-12 to 0 by 1)

gridattrs=(color=white)

labelattrs=(color=black family=Arial size=11 weight=bold)

valueattrs=(color=black family=Arial size=10);

run;

***/* code to run the LMM on delta mrdt (one example is provided referring to the comparison between respiratory infections and noninfectious respiratory diseases) */***

data a;

set deltamrdt;

w=1/(se_delta_mrdt*se_delta_mrdt); /* computing the weights */

if cause = 'respiratory infections - respiratory diseases'; /* selecting the causes of death */

proc mixed data=a covtest;

class country year(ref='1955') ;

model percent_change_mrdt = delta_a year / solution cl outpm=pred;

random intercept / subject=country;

weight w;

lsmeans year/diff=all adjust=bon;

run;

proc sort ;

by year pred;

run;

proc sort ;

by pred;

run;

ods listing gpath="/home/gabriele.sorci/" dpi=600; /* code to draw the graph */

ods graphics / reset

imagename="figure"

outputfmt=png

width=8in

height=6in;

ods graphics / reset

imagename="figure"

outputfmt=png

width=8in

height=6in;

title "respiratory infections - noninfectious respiratory diseases" ;

proc sgpanel data=pred noautolegend;

styleattrs

datasymbols=(CircleFilled)

datacontrastcolors=(bip bippk )

datacolors=(bip bippk );

panelby year / columns=2 rows=1;

scatter x=delta_a y=percent_change_mrdt / group=year

markerattrs=(size=5px)

jitter

jitterwidth=0.5;

series x=delta_a y=Pred/group=year lineattrs=( thickness=2 pattern=solid);

band x=delta_a upper=Upper lower=Lower/group=year fill transparency=0.5;

rowaxis

label="percent change mortality rate doubling time"

values=(-1 to 1 by 0.2)

gridattrs=(color=white)

labelattrs=(color=black family=Arial size=11 weight=bold)

valueattrs=(color=black family=Arial size=10);

colaxis

label="Δlog adult mortality rate at centered age"

values=(-2 to 2 by 0.5)

gridattrs=(color=white)

labelattrs=(color=black family=Arial size=11 weight=bold)

valueattrs=(color=black family=Arial size=10);

run;

quit;

***/* code to compute mean age at death and lifespan equality */***

proc iml;

use deaths;

read all var {age deaths} into X;

close deaths;

age = X[,1];

d	= X[,2];

N = sum(d);

mu = sum(age # d) / N;

nAge = nrow(age);

/* Expand age vectors into matrices */

age_i = repeat(age, 1, nAge); /* rows */

age_j = repeat(age`, nAge, 1); /* columns */

/* Pairwise absolute differences */

diff = abs(age_i - age_j);

/* Outer product of deaths */

w = d * d`;

/* Double sum */

num = sum(w # diff);

G = num / (2 * mu * N##2);

lifespan_equality = 1 - G;

print mu[label="Mean age at death"]

G[label="Gini"]

lifespan_equality[label="Lifespan equality (1-G)"];

quit;

***/* code to perform the Shapely decomposition */***

/* =============================================================== */

/* FULL DISTRIBUTION SHAPLEY DECOMPOSITION	*/

/* Factors: */

/*	1) Child mass (pre-adult mortality)	*/

/*	2) Gompertz level (b0)	*/

/*	3) Gompertz slope (b1)	*/

/* Works for all countries and causes	*/

/* Requires: */

/*	deaths_input	(country age deaths cause year) */

/*	gompertz_parms (country cause b0 b1 year)	*/

/* =============================================================== */

%let year0 = 1955;

%let year1 = 2020;

%let adult_min = 30;

%let omega	= 110;

%let center	= 57.5;

/* =============================================================== */

/* STEP 1 — CHILD DISTRIBUTION	*/

/* =============================================================== */

proc sql;

create table child_dist as

select country,

cause,

year,

age,

deaths / sum(deaths) as f_child

from deaths_input

where age < &adult_min

group by country, cause, year;

quit;

/* =============================================================== */

/* STEP 2 — ADULT MASS BY YEAR	*/

/* =============================================================== */

proc sql;

create table adult_mass as

select country,

cause,

year,

sum(case when age >= &adult_min then deaths else 0 end)

/ sum(deaths) as p_adult

from deaths_input

group by country, cause, year;

quit;

proc sql;

create table mass_wide as

select country,

cause,

max(case when year=&year0 then p_adult end) as p0,

max(case when year=&year1 then p_adult end) as p1

from adult_mass

group by country, cause;

quit;

/* =============================================================== */

/* STEP 3 — WIDE GOMPERTZ PARAMETERS	*/

/* =============================================================== */

proc sql;

create table parm_wide as

select country,

cause,

max(case when year=&year0 then b0 end) as b0_0,

max(case when year=&year1 then b0 end) as b0_1,

max(case when year=&year0 then b1 end) as b1_0,

max(case when year=&year1 then b1 end) as b1_1

from gompertz_parms

group by country, cause;

quit;

/* =============================================================== */

/* STEP 4 — BUILD 8 SHAPLEY SCENARIOS (3 FACTORS)	*/

/* =============================================================== */

data scenarios;

merge mass_wide parm_wide;

by country cause;

array p[2] p0 p1;

array b0[2] b0_0 b0_1;

array b1[2] b1_0 b1_1;

do i=0 to 1; /* mass */

do j=0 to 1; /* b0	*/

do k=0 to 1; /* b1	*/

scen = cats(i,j,k);

p_use = p[i+1];

b0_use = b0[j+1];

b1_use = b1[k+1];

output;

end;

end;

end;

keep country cause scen p_use b0_use b1_use;

run;

/* =============================================================== */

/* STEP 5 — SIMULATE ADULT DISTRIBUTION FOR EACH SCENARIO	*/

/* =============================================================== */

proc sort data=scenarios;

by country cause scen;

run;

data adult_dist;

set scenarios;

by country cause scen;

retain S;

do age=&adult_min to &omega;

if first.scen then S=1;

agec = age - &center;

mu = exp(b0_use + b1_use*agec);

d = S*(1-exp(-mu));

S = S*exp(-mu);

output;

end;

keep country cause scen age d p_use;

run;

/* Normalize adult part */

proc sql;

create table adult_norm as

select country,

cause,

scen,

age,

p_use*(d / sum(d)) as f_adult

from adult_dist

group by country, cause, scen;

quit;

/* =============================================================== */

/* STEP 6 — MERGE CHILD + ADULT TO GET FULL DISTRIBUTION	*/

/* =============================================================== */

proc sql;

create table full_dist as

select a.country,

a.cause,

a.scen,

a.age,

a.f_adult as f

from adult_norm as a

union all

select c.country,

c.cause,

s.scen,

c.age,

(1 - s.p_use)*c.f_child as f

from child_dist as c

inner join scenarios as s

on c.country=s.country

and c.cause=s.cause

and c.year = (case substr(s.scen,1,1)

when '0' then &year0

else &year1 end)

;

quit;

/* =============================================================== */

/* STEP 7 — COMPUTE TOTAL EQUALITY FOR EACH SCENARIO	*/

/* =============================================================== */

proc sql;

create table mean_full as

select country,

cause,

scen,

sum(age*f) as mean_age

from full_dist

group by country, cause, scen;

quit;

proc sql;

create table abs_full as

select a.country,

a.cause,

a.scen,

sum(a.f*b.f*abs(a.age-b.age)) as num

from full_dist as a

inner join full_dist as b

on a.country=b.country

and a.cause=b.cause

and a.scen=b.scen

group by a.country, a.cause, a.scen;

quit;

proc sql;

create table equality_full as

select a.country,

a.cause,

a.scen,

1 - (a.num / (2*b.mean_age)) as equality

from abs_full as a

inner join mean_full as b

on a.country=b.country

and a.cause=b.cause

and a.scen=b.scen;

quit;

/* =============================================================== */

/* STEP 8 — EXACT 3-FACTOR SHAPLEY DECOMPOSITION	*/

/* =============================================================== */

proc sql;

create table shapley as

select country,

cause,

max(case when scen='000' then equality end) as E000,

max(case when scen='100' then equality end) as E100,

max(case when scen='010' then equality end) as E010,

max(case when scen='001' then equality end) as E001,

max(case when scen='110' then equality end) as E110,

max(case when scen='101' then equality end) as E101,

max(case when scen='011' then equality end) as E011,

max(case when scen='111' then equality end) as E111

from equality_full

group by country, cause;

quit;

/* Shapley weights for 3 factors */

data final;

set shapley;

delta_total = E111 - E000;

phi_mass =

(1/3)*(E100-E000)

+(1/6)*(E110-E010)

+(1/6)*(E101-E001)

+(1/3)*(E111-E011);

phi_b0 =

(1/3)*(E010-E000)

+(1/6)*(E110-E100)

+(1/6)*(E011-E001)

+(1/3)*(E111-E101);

phi_b1 =

(1/3)*(E001-E000)

+(1/6)*(E101-E100)

+(1/6)*(E011-E010)

+(1/3)*(E111-E110);

run;

/* Consistency check */

proc means data=final sum;

var delta_total phi_mass phi_b0 phi_b1;

run;
